# Spousal violence and its determinants among married adolescent girls in Upper Egypt

**DOI:** 10.1186/s42506-020-00057-8

**Published:** 2020-10-13

**Authors:** Amira F. El-Gazzar, Mirette M. Aziz, Heba M. Mohammed, Omaima Elgibaly, Manal M. Darwish

**Affiliations:** Public Health & Community Medicine, Faculty of Medicine, Assiut University, Assiut, 71515 Arab Republic of Egypt

**Keywords:** Spousal violence, Upper Egypt, Early marriage, Married adolescent girls

## Abstract

**Introduction:**

In Egypt, many girls are still married before the age of 18, which is a fundamental violation of the girls’ human rights. Early marriage is associated with an alarmingly elevated risk of all types of intimate partner violence that have various negative consequences. The purpose of this study was to identify the predictors of exposure to spousal violence among the early married girls in rural Upper Egypt.

**Methods:**

A household survey was carried out and covered 23 villages in Assiut and Sohag governorates reaching to a sample of 729 married girls before the age of 20. Listing and enumeration of 4 districts was done to identify the study participants. Data was collected by personal interviews using a structured questionnaire. Bivariate and stepwise regression analyses were performed to identify the predictors of exposure to spousal violence.

**Results:**

It was found that 15.2% of the study participants were exposed to physical violence while 17.8% were exposed to sexual violence and 7.3% were exposed to both types. Girls married before the age of 18 were more exposed to spousal violence. Stepwise regression analysis found that girls’ acceptance to get married was a protective factor against exposure to physical (*β* = − 1.07, OR 0.34) and sexual (*β* = − 0.68, OR 0.51) violence. The perceived attitude of husbands and mothers-in-law about considering wife beating “a husband's right” was found to be a risk factor of exposure to physical and sexual violence. Longer duration till the first pregnancy was also associated with more exposure to sexual violence (*β* = 0.04, OR 1.04).

**Conclusion:**

Married adolescent girls (MAGs) are highly exposed to physical and sexual violence. This is mainly due to ignoring girls’ preference to postpone their marriage, cultural concepts of accepting violence against women, and low sexual satisfaction. This study shows that most determinants of spousal violence were related to culture issues. Identifying these determinants is required to combat such a crucial public health problem that has serious consequences on adolescent health.

## Introduction

In Egypt, despite the legislation of the Child Law of 2008 which sets the minimum age of marriage at 18 years for females and males, many girls are still married before the age of 18, which is a fundamental violation of the girls’ human rights [1].

The 2014 Survey of Young People in Egypt (SYPE) revealed that 1.7% of married female youth aged 25–29 in 2014 had been married before age 15, 4.2% before age 16, and 21.1% before age 18 which is the legal age of marriage in Egypt. Moreover, child marriage remains to be common in some parts of the country; 33.3% of married female youth aged 25–29 residing in rural Upper Egypt were married before age 18, compared to just below 10% of those in the Urban Governorates and urban Lower Egypt [2].

Early marriage has been associated with withdrawal from school and low opportunity for employment and social mobility [3, 4]. Hence, those girls usually enter marriage with low levels of education and limited knowledge and skills needed to negotiate adult marital roles [5].

Furthermore, evidence suggests that early marriage is associated with an elevated risk of intimate partner violence [6–8]. The 2014 Egypt Demographic and Health Survey (EDHS) showed that one in four married adolescent girls (MAGs) in Egypt (15–19 years) was ever exposed to some form of spousal violence while 5.5% were exposed to sexual violence [9]. A qualitative study among MAGs in three villages in Assiut revealed the presence of poor husband-wife communication, especially on matters related to achieving sexual satisfaction, thus leading to physical and sexual violence [10]. Moreover, a study in Iran has shown that spousal violence was significantly associated with low income, women age ≤ 20 years, unemployment, low education, being non-pregnant and non-house owners, and rural residence [11].

Spousal violence is a major public health concern, since it is documented to be associated with poor health outcomes including injuries, sexual/reproductive health problems, anxiety, and depression [12].

While rural Upper Egypt has the highest levels of spousal violence [9], little is known about its predictors. The current study was designed and implemented to measure the extent of MAGs’ exposure to physical and sexual violence in rural Upper Egypt and identify more precisely the predictors of such exposure. Results of the study would help the policy makers to design evidence-based interventions and programs to ameliorate the negative effects of early marriage on their health and that of their children.

## Methodology

### Study design

Data was collected using a household survey of married adolescent girls (MAGs) in Assiut and Sohag governorates, which are among the poorest and most conservative governorates in Egypt [13, 14]. The survey covered 23 villages in Assiut and Sohag reaching to a sample of 729. Data was collected by personal interview using a structured questionnaire after taking the consent of the girls and their husbands (when required). The study was conducted in selected rural communities of Assiut and Sohag governorates that were nominated by the health directorates in the two governorates based on the high prevalence of early marriage.

### Recruitment of the study population

Married adolescent girls (under 20 years) who had been married for at least 3 months at the time of data collection were invited to participate in the study. Enumeration of all households in the selected communities and listing married inhabitants of these households were conducted by a trained team to identify the households with at least one MAG. The team was escorted by community outreach workers to facilitate their entry into the houses and alleviate the tension of the locals. The heads of the households were approached by the members of the enumeration and listing team asking them to list all married women in the household together with their ages and duration of marriage and data was validated using the identity cards of the listed married women. The listing process yielded only 730 MAGs, who were all included in the study rather than applying random selection. The interviews were conducted at a quiet area at the MAG’s house or a place of her choice (e.g., health center) and lasted for about 1 h. Field training of data collectors involved pilot testing of the instrument among 20 MAGs in two villages that were not selected for the study.

### Questionnaire

All the study participants were interviewed using a structured questionnaire divided into sections covering all the variables that we hypothesized to be associated with exposure to spousal violence. The first section of the questionnaire detailed the socio-demographic characteristics. The second section assessed the practices related to asking the girls’ opinion before marriage, pre-marital counseling, marital sexual relation, pregnancy, and delivery. The third section explored the social support to the MAGs by their husbands and mothers-in-law including her social mobility and participation in decision-making as well as the attitudes of husbands and mothers-in-law about violence as reported by the MAGs. The fourth section discussed the exposure of MAGs to physical and sexual violence in the year preceding data collection.

### Statistical analysis

Data were analyzed using SPSS Statistics for Windows (version 21.0, NY). Descriptive analysis was performed for the participants’ socio-demographic data. Frequencies of exposure to physical and sexual violence were calculated.

The MAGs were considered to be exposed to physical violence if responded “Yes” to any of the following: being slapped or having something thrown at you that could hurt you; being pushed or shoved; being hit with a fist or something else that could hurt; being kicked, dragged, or beaten up; being choked or burnt on purpose; and/or being threatened with, or actually, having a gun, knife, or other weapon used on you. While the MAGs were considered to be exposed to sexual violence if responded by “Yes” to any of the following: practicing sex against will, having a sexual relation when the husband was drunk or drugged, and being forced to do sexual acts that she was not comfortable with.

*Decision-making index* was created by using 10 questions expressing the girls’ participation in taking different decisions. The response was given a score of “1” if the girl participated in the decision and “0” if she did not. *Social mobility index* was also created by using variables expressing the free mobility of the MAGs; 12 questions were used including the ability of the MAG to go to different places unescorted such as the doctor, family house, friend’s house, and market. The score “1” expressed the ability to go to the place and “0” as inability to go. Cronbach’s alpha for both scales was calculated as 0.7 and 0.6 respectively.

In the previous indices, an additive score was computed by summing the scores and the mean score percent was calculated as score/optimal score × 100.

Reported sexual satisfaction was assessed using a single question which asks about the overall sexual satisfaction and was graded along a Likert scale ranging from 1 to 5, where the score “1” denoted the least sexual satisfaction and the score “5” denoted the highest satisfaction.

*T* test and chi-square test were used to identify the significant factors associated with exposure to physical and sexual violence. All significant variables on bivariate analysis were considered for inclusion in the backward regression analysis. *P* value < 0.05 was considered as significant.

## Results

Table 1 shows the socio-demographic characteristics of the studied sample. The survey included 729 (324 in Assiut and 405 in Sohag) married girls. The majority got married (89.7%) under the age of 18. In both governorates, married girls shared similar socio-demographic characteristics. The mean age of girls was 18 years, and the husbands' mean age was 26.86 ± 6.4 years in Assiut and 25.69 ± 4.5 in Sohag with an average age difference between the husband and wife of 7–8 years. Almost all girls in both governorates were housewives. Consanguineous marriages were high among the studied group (59% and 67% in Assiut and Sohag respectively). Overall, the educational level among Sohag girls was less than that among Assiut girls. The husband’s educational status was better than their spouses. The mean duration between marriage and first pregnancy was 4.48 months for the total sample.

**Table 1:** Socio-demographic characteristics of the married adolescent girls (MAGs), Upper Egypt

Variable	Assiut	Sohag	***Total***
	***N*** = 324 (%)	***N*** = 405 (%)	***N*** = 729 (%)
**Age at marriage (years)**			
**<** 18	293 (90.4)	361 (89.1)	654 (89.7)
≥ 18–20	31 (9.6)	44 (10.9)	75 (10.3)
Mean ± SD	16.67 ± 1.40	16.47 ± 1.64	16.6 ± 1.5
**Age at time of study**			
Mean ± SD	18.33 ± 1.32	18.47 ± 1.42	18.4 ± 1.38
Duration of marriage (months)			
Mean ± SD	19.2 ± 13.9	23.5 ± 17.0	21.6 ± 15.9
**Girls educational level**			
Illiterate	21 (6.5)	81 (20.0)	102 (13.9)
Basic education	89 (27.5)	143 (35.3)	232 (31.8)
Secondary education	121 (37.3)	127 (31.4)	248 (34.0)
> Secondary education	93 (28.7)	54 (13.3)	147 (20.3)
**Work status**			
Work for cash	6 (1.9)	2 (0.5)	7 (1.0)
Not working	318 (98.1)	403(99.5)	721 (99.0)
**Parity**			
0	180 (55.6)	217 (53.6)	397 (54.5)
1	119 (36.7)	133 (32.8)	252 (34.6)
2	25 (7.7)	46 (11.4)	71 (9.7)
3	0 (0)	9 (2.2)	9 (1.2)
**Duration of marriage till first pregnancy** ***n*** **= 521** (247 Assiut, 274 Sohag)			
Mean ± SD	4.05 ± 5.9	5.2 ± 8.1	4.6 ± 7.1
**Husband age**			
Mean ± SD	26.86 ± 6.4	25.69 ± 4.5	26.2 ± 5.4
**Husband education**			
Illiterate	17 (5.3)	40 (9.9)	57 (7.8)
Basic education	44 (13.6)	76 (18.8)	120 (16.5)
Secondary education	26 (8.0)	52 (12.8)	78 (10.7)
> Secondary education	237 (73.1)	237 (58.5)	474 (65.0)
**Consanguineous marriage**			
Yes	189 (58.3)	260 (64.2)	449 (61.6)
No	135 (41.7)	145 (35.8)	280 (38.4)
**Co-residence**			
Lives with husband alone	52 (16.0)	34 (8.4)	86 (11.8)
Lives with others	272 (84.0)	371 (91.6)	643 (88.2)

The norm for the studied MAGs in the early years of their marriage is to live in an extended family (the husband’s family): 84% and 91.6% in Assiut and Sohag respectively.

Table 2 shows the percentage of exposure of the studied MAGs to physical and sexual violence in the last year. Exposure to physical violence was 15.2% (21.0% in Assiut and 10.6% in Sohag), while exposure to sexual violence was slightly higher 17.8% (24.7% in Assiut and 12.3% in Sohag).

**Table 2:** Exposure of the married adolescent girls (MAGs) to physical and sexual violence, Upper Egypt

Types of violence	Assiut	Sohag	***N*** = 729 (%)
	***N*** = 324 (%)	***N*** = 405	
**Physical violence**			
Exposed	68 (21.0)	43(10.6)	111 (15.2)
Non-exposed	256 (79.0)	362 (89.4)	618 (84.8)
**Sexual violence**			
Exposed	80 (24.7)	50 (12.3)	130 (17.8)
Non-exposed	244 (75.3)	355 (87.7)	599 (82.2)
**Exposed to both physical and sexual violence**	35 (10.8)	18 (4.4)	53 (7.3)

Table 3 shows that exposure of MAGs to physical and sexual violence was significantly related to living with the husband alone, longer duration of marriage, and longer duration till the first pregnancy, while acceptance of marriage timing and sexual satisfaction were significantly associated with less exposure to both types of violence. It also shows that the agreement of the husbands and mothers-in-law that wife beating is a “husband right” was significantly associated with more exposure to physical and sexual violence. Moreover, sexual violence was significantly associated with marriage at age less than 18 while receiving pre-marital counseling was protective.

**Table 3:** Factors affecting exposure of married adolescent girls (MAGs) to physical and sexual violence, Upper Egypt

Variables	Physical violence		Sexual violence	
	Exposed	Non-exposed	Exposed	Non-exposed
**Age at marriage (years)**				
Below 18	101 (15.4)	553 (84.6)	124 (19.0)*	530 (81.0)
18 and above	10 (13.3)	65 (86.7)	6 (8.0)	69 (92.0)
**Girls’ education**				
Illiterate	16 (76.2)	5 (23.8)	7 (33.3)	14 (66.7)
Lower than secondary education	169 (80.5)	41 (19.5)	58 (27.6)	152 (72.4)
Secondary education and above	71 (76.3)	22 (23.7)	15 (16.1)	78 (83.9)
**Parity**				
Mean ± SD	1.1 ± 0.7	1.0 ± 0.4	1.2 ± 0.6	1.0 ± 0.5
**Husband’s age**				
Mean ± SD	26.6 ± 3.9	26.9 ± 6.8	26.0 ± 4.4	27.2 ± 6.9
**Husband’s education**				
Illiterate	6 (35.3)	11	5 (29.4)	12 (70.6)
Lower than secondary education	19 (27.1)	51	23 (32.9)	47 (67.1)
Secondary education and above	43 (18.1)	194	52 (21.9)	185 (78.1)
**Consanguineous marriage**				
**Yes**	40 (21.2)	149 (78.8)	50 (26.5)	139 (73.5)
**No**	27 (20.6)	104 (79.4)	29 (22.1)	102 (77.9)
**Co-residence**				
Living with husband alone	26 (30.2)***	60 (69.8)	25 (29.1)**	61 (70.9)
Living with others	85 (13.2)	558 (86.8)	105 (16.3)	538 (83.7)
***Pre-marital factors***				
**Girl’s opinion about timing of marriage**				
Agree	61 (11.5)***	471 (88.5%)	75 (14.1)***	457 (85.9)
Disagree	50 (25.4)	147 (74.6)	55 (27.9)	142 (72.1)
**Receiving pre-marital counseling**				
Received	27 (12.2)	194 (87.8)	27 (12.2)**	194 (87.8)
Not received	84 (16.5)	424 (83.5)	103 (20.3)	405 (79.7)
***Marital factors***				
**Duration of marriage (months)**				
Mean ± SD	25.3 ± 17.4**	20.9 ± 15.5	25.1 ± 16.8**	20.8 ± 15.6
**Duration of marriage till the first pregnancy (months)**				
Mean ± SD	6.2 ± 8.1*	4.4 ± 6.9	6.97 ± 9.8***	4.1 ± 6.3
**Perceived husband's attitude towards wife beating**				
Agree	91 (20.8)***	346 (79.2)	92 (21.1)**	345 (78.9)
Disagree	20 (6.8)	272 (93.2)	38 (13.0)	254 (87.0)
**Perceived mother-in-law's attitude towards wife beating**				
Agree	82 (18.8)**	355 (81.2)	91 (20.8)*	346 (79.2)
Disagree	29 (9.9)	263 (90.1)	39 (13.4)	253 (86.6)
**Sexual satisfaction score**				
Mean ± SD	3.96 ± 0.9***	4.3 ± 0 .5	4.1 ± 0.89**	4.31 ± 0.53
**Social mobility score**				
Mean ± SD	0.8 ± 1.1*	0.6 ± 0.9	0.7 ± 0.96	0.6 ± 0.97
**Decision-making score**				
Mean ± SD	4.7 ± 2.2	4.6 ± 2.1	4.5 ± 2.5	4.7 **±** 2.04

*P value < 0.05, **P value < 0.01, ***P value < 0.001

Table 4 shows that by backward regression analysis, it was found that acceptance of marriage timing, living with other family members, and sexual satisfaction were the significant protective factors against exposure to physical and sexual violence. Moreover, considering wife beating as “a husband right” was associated with more exposure to physical and sexual violence, and longer duration till first pregnancy was associated with more exposure to sexual violence.

**Table 4:** Predictors of exposure of married adolescent girls (MAGs) to physical and sexual violence, Upper Egypt

Predictors	Exposure to physical violence			Exposure to sexual violence		
	***β***	Odds ratio	CI	***β***	Odds ratio	CI
Perceived husband attitude about practicing physical violence	0.80*	2.24	1.5–4.7	0.82**	2.270	1.4–8.2
Co-residence	− 0.82*	0.46	0.1–0.78	− 0.75*	0.48	0.21–0.57
Girl's opinion about marriage timing	− 1.07***	0.34	0.13–0.61	− 0.68**	0.51	0.3–0.7
Reported sexual satisfaction	− 0.77***	0.46	0.31–0.88	− 0.39*	0.68	0.31–0.85
Duration till first pregnancy	0.02	1.03	0.97–1.1	0.04**	1.04	1.01–1.08
Duration of marriage	0.01	1.01	0.98-1.0	0.06	1.0	0.9–1.0
Perceived mother-in-law's attitude about practicing physical violence	0.07	0.93	0.71–1.2	0.13	1.14	0.51–2.58
Social mobility score	0.02	1.02	0.95–1.1	− 0.009	0.99	0.92–1.06
Age at marriage (below 18)	–	–	–	0.037	1.03	0.22–4.7

*P value < 0.05, **P value < 0.01, ***P value < 0.001

## Discussion

In Egypt, 30% of ever-married women aged 15–49 reported having been ever subjected to at least one episode of physical, sexual, and/or emotional violence inflicted by their current or most recent spouse; however, sexual violence was only reported by 4% of the participants [9]. In Alexandria, a cross-sectional survey found that more than three-quarters of the participants (77%) reported experiencing spousal violence. Emotional violence was the most commonly reported form (71.0%), followed by physical (50.3%) and sexual violence (37.1%) [15].

Despite the fact that all married women from all age groups are at risk of spousal violence, married adolescent girls are at much higher risk of exposure to violence and its consequences [16]. In Egypt, one in every four adolescent married girls has been ever exposed to some form of spousal violence while 5.5% have been exposed to sexual violence [9].

Early marriage is still common in Upper Egypt, and most (89.7) of the participants in this study got married under the age of 18. Since the legal age of marriage in Egypt is 18 years, girls who married before completing their 18th birthday were in discordance with the law and are more liable to be exposed to spousal violence. According to our study, 15.2% of the study participants were exposed to physical violence while 17.8% were exposed to sexual violence in the last year (prior to the year of data collection). This percentage would have been definitely higher if we asked about exposure throughout the marital life.

Our study showed that spousal violence was associated with various cultural factors. In many cultures, it is acceptable for men to control their wives’ behavior and those women who refuse may be punished. Studies found that violence is frequently viewed as the husband’s right to discipline his wife [17]. Moreover, a study conducted between 1998 and 2001 from seven countries found that male acceptance of wife beating ranged from 26% in Kazakhstan to 56% in Turkey [18]. As shown in our study, male attitudes condoning partner violence is a crucial predictor of exposure to physical and sexual violence. This was consistent with various studies where men who justified wife beating and held more gender inequitable attitudes were more likely to abuse their wives [19, 20].

Taking the girls’ acceptance about the timing of getting married and the person she will marry not only is a human right, but also significantly affects her exposure to physical and sexual violence. Multivariable logistic regression analysis in our study showed that girls’ acceptance of the marriage timing was a significant protective factor against exposure to physical and sexual violence. This was consistent with a study conducted in Turkey to identify factors associated with domestic violence. The study found that the prevalence of domestic violence was significantly lower among women whose marriage was by mutual agreement [21]. This is probably due to the similarity between the Egyptian and Turkish culture, as usually the spouses will have no chance to know each other before getting married. Moreover, women especially MAGs that are forced to get married are mostly those without economic independence and thus are less able to overcome violence.

It is common in rural Upper Egypt for married couples to live in extended families specially married adolescent girls, 88.2% of our study sample lived in extended families. The extended family can significantly affect the marital relationship of a couple, especially in the early years of their marriage, and can play a positive as well as a negative role. Bivariate and multivariable logistic regression analyses in our study showed that exposure of MAGs to physical and sexual violence was significantly associated with living with the husband alone while living with other family members was a significant protective factor against exposure to physical and sexual violence. Consistent with our study, a qualitative study done in Pakistan and the UK indicated that the husband’s family can have a positive effect by minimizing conflict through offering the couple personal time and helping the wife to adjust to her new family and the wife’s family could contribute by helping their daughter to adjust to her new extended family [22].

Moreover, a study done in Jordan to identify the role of the extended family on women’s risk of intimate partner violence using mixed-methods study found that residence with the respondent’s in-laws act as a protective factor against conflict and intimate partner violence between a husband and wife [23]. However, some studies found that a wife’s in-laws act as instigators of conflict between husband and wife or a direct source of conflict with the wife and thus contributing to violence even if they do not live with or were near the couple [24, 25]. In addition, the focus group discussions (FGDs) of a study done in Jordan revealed that families were not always an effective source of assistance [23].

Sexual satisfaction is an integral part of marital life that affects the couple’s relationship [26]. Our study found a significant association between violence and reported sexual satisfaction; the higher the sexual satisfaction score of the MAG, the less was the exposure to physical and sexual violence. Previous studies have repeatedly shown this association between sexual satisfaction and spousal violence. Ulloa and Hammett [27] reported a negative correlation between intimate partner violence and satisfaction. Also, a study conducted in Iran showed a significant association between partner violence and all aspects of women’s sexual function [28].

The norm of Egyptian society is not postponing pregnancy of the first child especially in rural communities. Failure to conceive directly after marriage affects the psychological state of the husband, which leads to violence. Violence against women experiencing delayed pregnancy is an important health problem with serious consequences for their physical and mental health [29]. Our study revealed that longer duration till having the first baby was associated with more exposure to sexual violence.

Consistent with our study, a systematic review on the relationship between infertility, sub-fertility, and intimate partner violence found that infertility (inability to become pregnant)/sub-fertility (inability to maintain a pregnancy) are associated with violence in low- and middle-income countries [30]. Moreover, many studies found that fertility and sub-fertility could be risk factors for intimate partner violence [29–33]. Also, infertile women are exposed to higher levels of marital violence compared to fertile women [34].

### Limitations of the study

This large survey investigated an important and relatively unspoken issue but has some limitations; If husbands and mothers-in-law were interviewed, more insight on the problem would have been given, but this was very difficult in such conservative societies. Many families denied the presence of MAGs during the listing process since the legal age of marriage in Egypt is 18 years and families of MAGs who were below 18 years might face legal penalties if the case was reported to the authorities. To overcome this challenge, the enumeration and listing team members were escorted by outreach workers from the local communities to establish rapport with the families and enable the team to clarify that the collected data will be used for scientific purpose, will never be shared with the authorities, and that the filled questionnaires will have no identifiers, will not be linked to the developed lists, and the results will be presented in an aggregate form. Moreover, the data collectors were provided with identification cards affiliated to Assiut University and copies of the Central Agency for Public Mobilization and Statistics (CAPMAS) approval of the study to assure them that they were only research-participants.

## Conclusion

Girls married before the age of 18 are exposed to spousal physical and sexual violence. Taking the girls’ opinion at the time of marriage and obtaining their acceptance are protective against exposure to physical and sexual violence. Moreover, cultural beliefs that consider spousal violence acceptable defiantly increase the risk of girls being exposed to violence.

In rural communities, young girls that lived with their husbands alone were more exposed to violence, which could be attributed to their inability to be totally independent. This study clearly illustrates the need for enforcement of laws as well as raising the community awareness to delay the age of marriage and respect “the girl’s will” to delay her marriage until she is more autonomous, able to participate in decision-making, and more capable of bearing other marital responsibilities. In addition, mass media campaigns and public educational sessions directed towards the elimination of gender discrimination should be widely used in order to change the cultural concepts that consider wife beating a “husband right.” Incorporation of sexual counseling within the MCH services and making it available for couples is required to improve sexual satisfaction, conflict resolution skills, and other life skills.

## Data Availability

Available on reasonable request.

## Abbreviations

MAGs: Married adolescent girls
